# Development and optimizing a simple and cost-effective medium for in vitro culture of *Plasmodium berghei*-ANKA strain with conserving its infectivity in BALB/c mice

**DOI:** 10.1186/s13104-022-05946-z

**Published:** 2022-02-15

**Authors:** Haleh Hanifian, Mehdi Nateghpour, Afsaneh Motevalli Haghi, Aref Teimouri, Sepand Razavi, Leila Fariver

**Affiliations:** 1 Department of Medical Parasitology and Mycology, School of Public Health, Tehran University of Medical Sciences, Tehran, Iran; 2 Hadith and Medicine, Research Centre of Quran, Tehran University of Medical Sciences, Tehran, Iran; 3 Department of Parasitology and Mycology, School of Medicine, Shiraz University of Medical Sciences, Shiraz, Iran

**Keywords:** Malaria, *Plasmodium berghei*, In vitro culture, BALB/c mice

## Abstract

**Objectives:**

The current culture system for *P. berghei* still requires modifications in consistency and long-term maintenance of parasites considering their pathogenicity in culture media. Therefore, this study designed to further improvement of culture conditions and designing a cost-effective culture medium with minimum changes in pathogenicity for in vitro culture of *P. berghei.*

**Results:**

Results indicated that the rate of parasitaemia in our modified method remained statistically stable between days one to seven (*P* = 0.07). The current modified cultivation method was more efficient in maintaining of parasites for further days. Furthermore, in current method the stability of parasitaemia rate during day1 to day7 was in better rate compared to that in Ronan Jambou et al. and the differences between two methods were statistically significant (*P* = 0.001). The virulence of cultivated parasites in our modified method remained similar to frozen stock parasites as positive control group. No significant differences were seen in survival time between two groups of mice those were infected with either cultivated parasites or stock freeze parasites (*P* = 0.39) with the mean survival time of 20.83 ± 3.84 and 19.66 ± 1.21 days, respectively. Herein, we achieved a simple, cost-effective and applicable technique for culture of *P. berghei*.

**Supplementary Information:**

The online version contains supplementary material available at 10.1186/s13104-022-05946-z.

## Introduction

Malaria has been considered as a significant threat to health systems throughout the world for decades and presently, one third of the world's population are at risk of the infection [1, 2]. Global attempts to manage the disease, led to documented decrease in levels of morbidity and mortality due to malaria and nowadays the disease is approaching some levels of control worldwide [3, 4]. *Plasmodium falciparum*, *P. vivax*, *P.* *ovale*, *P.* *malariae* and *P.* *knowlesi* are the most important causes of malaria, however, *P. falciparum* is considered as the deadliest species, accounting for an estimated 99.7% of malaria cases in Africa according to WHO report in 2018 [2, 5, 6]. Various procedures are conducted to control the malaria infection including diagnosis methods, treatment of patients, preparation of novel medications as well as control of vectors [7, 8]. One of the most important factors in performing control programs is to understand the biology of *Plasmodium* parasites in more details. However, the most imperative problem in conducting biological studies on malaria parasites is the cultivation and purification of the parasites [8]. Cultivating human malaria parasites (*P. falciparum* for instance) with gametocyte maturation stages, can lead to establishing of parasite cycle in nature if Anophel is available. Therefore, it is better to use non-human malaria parasites species but, phylogenetically close to human species in cultivation procedures [9, 10]. *Plasmodium berghei* is an African murine malaria parasite isolated by Vincke and Bafort in Katanga (PbNK for New York-Katanga) and in Kasapa (PbANKA for Antwerpen-Kasapa) and appropriate substitute model for studying *Plasmodium* species in both in vitro and in vivo investigations [11–14]. The first attempts were made to cultivate *P. berghei* on cell culture media conducted by Mons et al. in 1983 [15]. In vitro culture of *P. berghei* ANKA with maintaining infectivity of mouse erythrocytes inducing cerebral malaria described by Ronan Jambou et al. [11]. However, employing high quality culture media is expensive, sensitive and also due to variations in pathogenicity of parasites, cannot be used in pharmaceutical and similar studies. Moreover, the most notable issue in performing *P. berghei* cultivation is the necessity of completing several passages which may lead to a considerable reduction in pathogenicity of parasites and serious problems in biological studies, consequently [15, 16]. Therefore, the aim of the present study was to design a simple and cost-effective medium to maintain parasites for longer time with a mild rate of reduction during days and considering the changes in pathogenicity of cultivated parasites.

## Main text

### Methods

#### Mice

Thirty-four male BALB/c mice, with 6–8 weeks age and weighing 25 ± 5 g, were purchased from the Animal Breeding Stock Facility, Razi Vaccine and Serum Institute of Iran, Karaj, Iran (24 mice for in vivo experiments and 10 mice for parasite cultivation stages). Animals were kept under the standard laboratory environmental conditions (light–dark cycle conditions, controlled temperatures of 22 °C ± 2 °C) with ad libitum foods and fresh drinking tap water. The mice were maintained at the animal house section of School of Public Health, Tehran University of Medical Sciences according to the Standard Guidelines for the Care and Use of Laboratory Animals and the Association for the Assessment and Accreditation of Laboratory Animal Care (AAALAC) [17]. The sample size considering 10 percent attrition was calculated according to the previously described method [18]. The animal to be euthanized were first anaesthetized with ketamine (75 mg/kg) given subcutaneously. Finally, cervical dislocation was used on mice to ensure that they have been properly euthanized. All procedure were performed to avoid causing unnecessary pain to animals based on the rules of animal care and use research.

#### Compounds

DMEM-F12, RPMI 1640 (containing HEPES and glucose) media and heat-inactivated fetal calf serum (FCS) were purchased from Gibco, Germany. Gentamicin, NaOH, L-glutamine, Hypoxanthine, Gelatin, Calcium Bicarbonate, Hydroethidine were purchased from Sigma-Alderich, Germany. Albumax II was provided from Pasteur Institute of Iran. *P. berghei* ANKA (PbA) strain was obtained from National Laboratory of Malaria, School of Public Health, Tehran University of Medical Sciences, Tehran, Iran.

#### Maintenance of in vitro culture

In this study, the ANKA strain was originated from stock collections and then propagated in BALB/c mice via intra-peritoneally (IP) inoculation of 10^6^ infected red blood cells (IRBCs) suspensions. Seven days after infection, when parasitaemia reached more than 10%, blood samples from infected mice were collected by cardiac puncture under the standard anaesthesia. All blood samples underwent the buffy coat removal process using routine phosphate buffered saline (PBS; pH = 7.4) washing method.

#### Maintenance of *P. berghei* using our modified technique

Starting this technique followed method described by Jensen and Trager [19] for *Plasmodium falciparum* cultivation with inspirations from protocol described by Ronan Janbou [11]. To prepare a complete culture medium (CCM), 500 mL RPMI 1640 medium (containing HEPES and glucose) supplemented with 10 mL NaOH 5 M, Hypoxanthine (0.027 gr dissolved in 5 mL DDW and 5µL NaOH 5 M),Gentamycin (0.025 gr dissolved in 2.5 mL DDW and 5µL NaOH) and 10% FBS. The cultivation was conducted in small petri-dishes those were placed in a candle jar and incubated at 34 °C in an incubator equipped with an orbital shaker (100 rpm) for 20 h. The media were daily centrifuged at 5000 g for 1 min and then suspended again with fresh medium and hematocrit was fixed at 10% using non-infected fresh red blood cells obtained from healthy mice. Fifty microliter of Albumax II was added every 3 days to culture media. At the next step, after successful cultivation of *P. berghi* with our modified method, we performed this method and method describe by Jambou et al., simultaneously [11].

#### Maintenance of* P. berghei *using Jambou et al. [11] technique

In this method culture media was comprised of RPMI1640 (containing HEPES, and L-Glutamine and Bicarbonate) (3/4), DMEM-F12 (1/4), Hypoxanthine 200 μM, Glucose 3 g/L, Gelatin 0.1%, Calcium 2 mM,,Choline 1 mM, Hydroethidine 200 μM and AlbuMAX II 0.5%. Culture media were stored at 32 °C and mice fresh and non-infected RBAs were added twice a week with the haematocrit rate at 2.5%. Culture flasks were kept closed in vertical position and gassed with a 5% O2, 5% CO2, and 90% N2 gas mixture with shaking at 100 rpm on an orbital shaker.

#### Microscopic examination

Monitoring of parasitemia and identification of the stage of the parasite in both techniques were carried out using thin blood smears staining with Giemsa stain following methanol fixation, and counting infected RBCs microscopically (Additional file 1: Figs. S1, S2). Rate of parasitemia was calculated by daily determination of the parasitaemia in culture, using thin blood Giemsa-stained smears. Parasites were counted on 100 fields at × 1000 magnification.

#### In vivo infectivity test

In vivo infectivity of the cultured parasites was evaluated by inoculating IRBCs in susceptible mice (BALB/c) using IP inoculation. The BALB/c mice were divided into 4 major groups including 6 mice in each group as follows: group 1 were infected with 10^6^ IRBCs of frozen stock parasites as positive control group; group 2 were infected with 10^6^ IRBCs but with those parasites that were cultivated in modified culture medium on the seventh days; group 3 received just PBS and group 4 remained uninfected as negative control group [20–22]. Mice of the first and second groups were bled from their tails one week after the infection, to evaluate the growth and mutilation of parasites. Moreover, in daily monitoring, the mortality rate was recorded for each group.

#### Statistical analysis

Statistical analysis was carried out using SPSS Software v.21 (IBM, Armonk, NY, USA). Statistical differences between groups were calculated using t-test with confidence intervals of 95%. Moreover, Kaplan–Meier method was used for comparison of the survival rates between the studied groups [23]. Data description was carried out by calculating frequencies and 95% confidence intervals. Differences were considered as significant when *P* ≤ 0.05. Results were reported as mean ± SD.

### Results

#### In vitro experiments

Parasitaemia rate in our modified technique assessed for 17 days, in three petri-dishes (Table 1). In this method, the number of merozoites and IRBCs were increased up to day 8 and eventually the parasitaemia rate was decreased to < 0.1% on day 16 (Table 1). The results indicated that, the rate of parasitaemia in modified method was not statistically different during day1 to day7 (*P* = 0.078). The modified cultivation method was more efficient in maintaining of parasites and stability of parasitaemia rate during day1 to day7 compared to that in Jambou et al. technique. The differences between two methods were statistically significant (*P* = 0.001) (Table 2).

**Table 1:** The rates of parasitaemia using modified technique during day1 to day17

Parasitaemia	Plate 1(%)	Plate 2 (%)	Plate 3 (%)	Mean ± SD
Day1	4.7	4.3	4.7	4.5 ± 0.2
Day 2	6.1	6.4	7.0	6.5 ± 0.3
Day 3	9.3	8.1	9.6	9.0 ± 0.6
Day 4	8.7	7.5	8.5	8.2 ± 0.5
Day 5	8.2	7.9	8.3	8.1 ± 0.1
Day 6	7.3	8.0	7.6	7.6 ± 0.3
Day 7	9.0	8.4	9.3	8.9 ± 0.3
Day 8	9.1	9.0	11.1	9.7 ± 0.9
Day 9	9.0	8.4	7.9	8.4 ± 0.3
Day 10	7.3	7.9	7.0	7.4 ± 0.3
Day 11	8.0	6.3	7.8	7.3 ± 0.7
Day 12	6.9	6.1	7.0	6.6 ± 0.4
Day 13	4.4	4.0	4.2	4.2 ± 0.1
Day 14	2.0	1.3	1.6	1.6 ± 0.2
Day 15	0.9	0.6	0.5	0.6 ± 0.01
Day 16	0.3	0.1	0.2	0.2 ± 0.01
Day 17	< 0.1	< 0.1	< 0.1

**Table 2:** Parasitaemia rate in the modified technique in comparison to Ronan Jambou et al. [11] method during day1 to day 7

Modified culture method			Method described by Ronan Jambou et al. [11]	
Mean ± SD		*P*- value	Mean ± SD	*P*- value
Day1	4.8 ± 0.2	0.078	4.6 ± 0.05	0.001
Day2	6.9 ± 0.4		4.5 ± 0.2	
Day3	8.3 ± 1.6		4.5 ± 0.05	
Day4	7.9 ± 0.3		3.1 ± 0.05	
Day5	8.1 ± 0.1		2.0 ± 0.0	
Day6	7.4 ± 0.2		0.5 ± 0.03	
Day7	6.2 ± 0.2		< 0.5	

#### In vivo infectivity

**Fig. 1:**
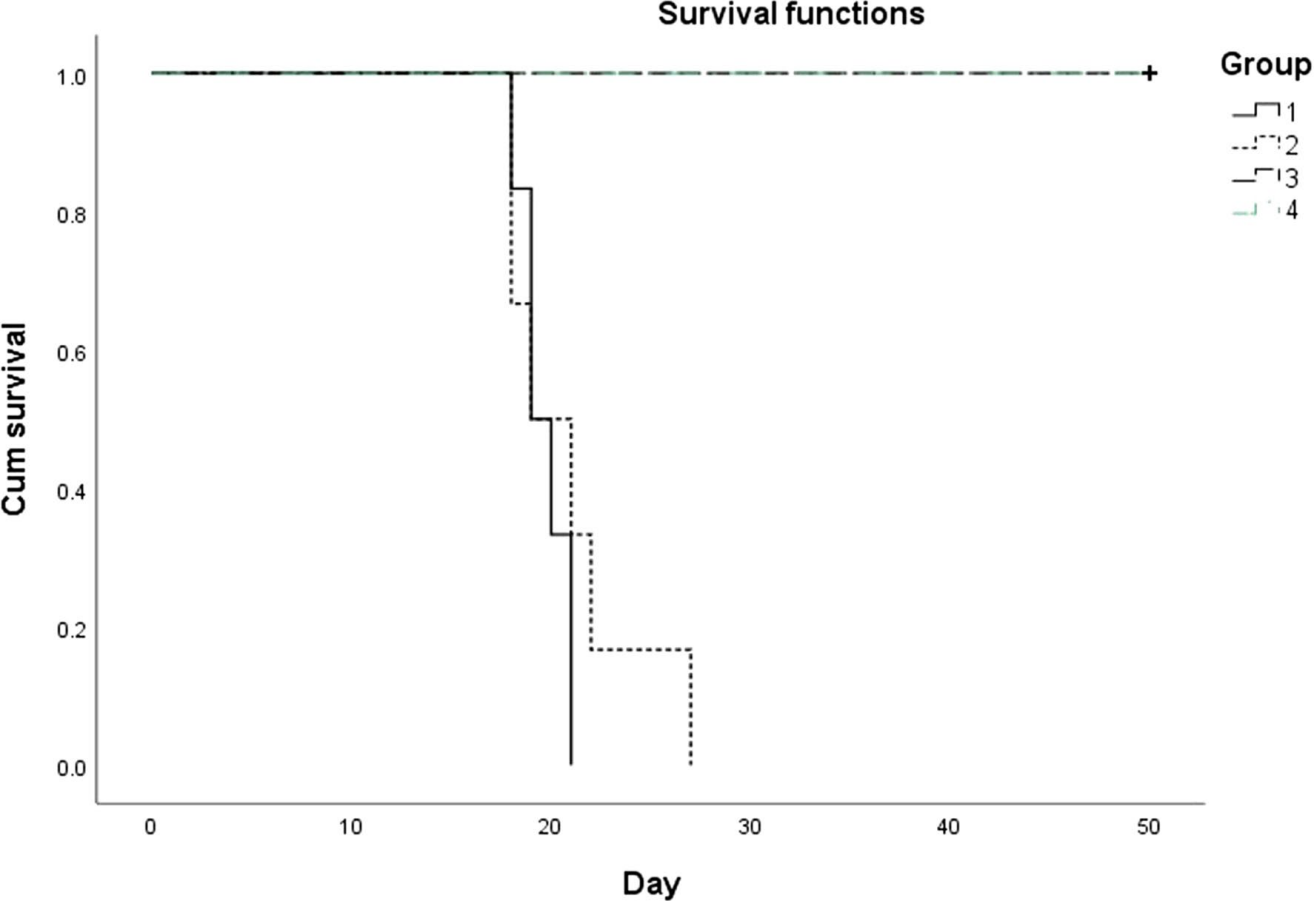
Survival rates of BALB/c mice inoculated with the freeze parasite (group 1), parasite collected from the culture medium on the seventh day (group 2), PBS (group 3), uninfected mice (group 4) (n = 6 per group)

The mean survival time of BALB/c mice in group 1 (inoculated with the freeze parasite) and group 2 (inoculated with parasite collected from the culture medium on the seventh day) include 19.66 ± 1.21 and 20.83 ± 3.84 days, respectively. All mice in group 1 died by day 21 but members of group 2 were survived by day 27. The third and fourth groups of non-infected mice were alive for up to 50 days (Fig. 1).

There was not any significant difference in survival time between group 1 and group 2 (*P* = 0.396). However, significant statically differences in survival time were observed among groups 1 and 2 against groups 3 and 4 (*P* = 0.001).

### Discussion

In this study, a modified cultivating technique for malaria parasites which was an adoption protocol of *P. falciparum* in vitro cultivation, was employed to cultivate *P. berghei*. In fact, the method used in this study was a composition method of was *P. berghei* and *P. falciparum* cultivation procedures that were established years ago [11, 24]. Although, ethical standards recommend the use of in vitro culture methods as an alternative to in vivo studies *Plasmodium* parasites, except for *P. falciparum*, are hard-growing microorganisms in culture medium and despite numerous studies, their cultivation has not been completely achieved, especially in continuous cultivations [25]. Many attempts have been made to cultivate different types of *Plasmodium*, and so far some species of them have achieved considerable success to preserve them, more or less, for long time with in vitro conditions. However, more studies are still crucial to attain faster, uncomplicated and inexpensive cultivation [26–28].

There are various limitations in cultivating *Plasmodium* parasites and the most challenging problems are the sequestration of RBCs, the lack of non-infected cells for penetration of parasites and also short life span of RBCs in in vitro techniques [28–30]. Changing the temperature and adding RBCs on a daily basis in this study, resulted to higher parasitaemia compared to previous culture media. For instance, the rate of parasitaemia in the medium described by Jambou et al. [11] was decreased sharply to less than 0.5% by day 7 (*P* = 0.001), whenever, in present modified method, the rate of parasitaemia was not significantly decreased until day 7 (*P* = 0.078). Moreover, the composition of culture media in present study was simpler and except essential components in *Plasmodium* spp. cultivation, RPMI for example, we need less agents to enhance the culture media.

In vitro culture of *P. falciparum* was first described by Jensen and Trager [19], Janse [11] and Hollingdale [31]. Long-term in vitro cultivation of *P. berghei* was claimed to be obtained by Ramaiya et al. [32] at 27 °C, and by Smalley [33] who achieved a low multiplication rate at 15 °C. However, at 37 °C, a decrease in parasite density was always observed as a consequence of the instability of the RBC. In present study due to some modifications including composition of culture media, reducing the temperature, employing a shaker and increasing hemoglobin rate, we could keep the parasitemia in a high level in first 7 days of culture process. Considering the necessity of further investigations, the results of current study and its important approaches in in vitro cultivation can be beneficial in malaria researches such as genetics studies, molecular and cellular biology and vaccine development.

One of the most important drawbacks of culture method, is the reduction of pathogenicity in cultivated parasites after several passages which may lead to a serious problems in biological studies. In a study that was conducted by Weiss and Degiusti [16], *P. berghei*, which normally kills mice within three weeks of infection, has been modified in virulence via serial passage through tissue culture. The parasite lost 80% of its infectivity against mice in comparison with control stock parasites. Indeed, those *P. berghei* parasites that were cultivated in our modified technique could maintain their effective virulence up to 7 days against the BALB/c subjected mice.

### Conclusions

In conclusion, this study shows that with a cost-effective culture medium and modified cultivation technique for *P. berghei*, accessing an applicable in vitro cultivation method with minimum changes in pathogenicity, is obtainable. Although all stages of human species of *Plasmodium* spp. have achieved different levels of success in cultivation methods, non-human species cultivation methods especially *P. berghei* and its erythrocytic stage has not changed significantly during recent years. Therefore, more investigations are needed to reach a reliable patent for alternation of *Plasmodium* species cultivation methods especially *P. berghei* for long-term culture procedures.

### Limitations

In this study, parasitaemia rate in our modified technique assessed for 17 days, in three petri-dishes. Culture groups could be more and categorized for in vitro experiment. Moreover, comparing between methods could be performed in multiple times.

## Supplementary Information

**Additional file 1: Figure S1**. Monitoring parasitemia in culture of P. berghei via thin blood smears staining with Giemsa stain. **Figure S2**. Monitoring parasitemia in culture of P. berghei via thin blood smears staining with Giemsa stain.

## Data Availability

All data generated or analyzed during this study are included in this published article. The raw data analyzed during the current study are publicly available via Figshare Repository (34).

## Abbreviations

WHO: World Health Organization
DMEM: Dulbecco's Modified Eagle Medium
RPMI: Roswell Park Memorial Institute Medium
FCS: Fetal Calf Serum
HEPES: N-2-Hydroxyethylpiperazine-N'-2-Ethanesulfonic Acid
IP: Intra-Peritoneally
IRBCs: Infected Red Blood Cells
PBS: Phosphate Buffered Saline
