# Supplementary material for: Development and optimizing a simple and cost-effective medium for in vitro culture of Plasmodium berghei-ANKA strain with conserving its infectivity in BALB/c mice
Source: BMC Res Notes. 2022 Feb 15;15:56. doi: 10.1186/s13104-022-05946-z (PMC8845400; doi:10.1186/s13104-022-05946-z)
Supplement: Supplementary file 1 — Additional file 1: Figure S1. Monitoring parasitemia in culture of P. berghei via thin blood smears staining with Giemsa stain. Figure S2. Monitoring parasitemia in culture of P. berghei via thin blood smears staining with Giemsa stain. [file 13104_2022_5946_MOESM1_ESM.docx]

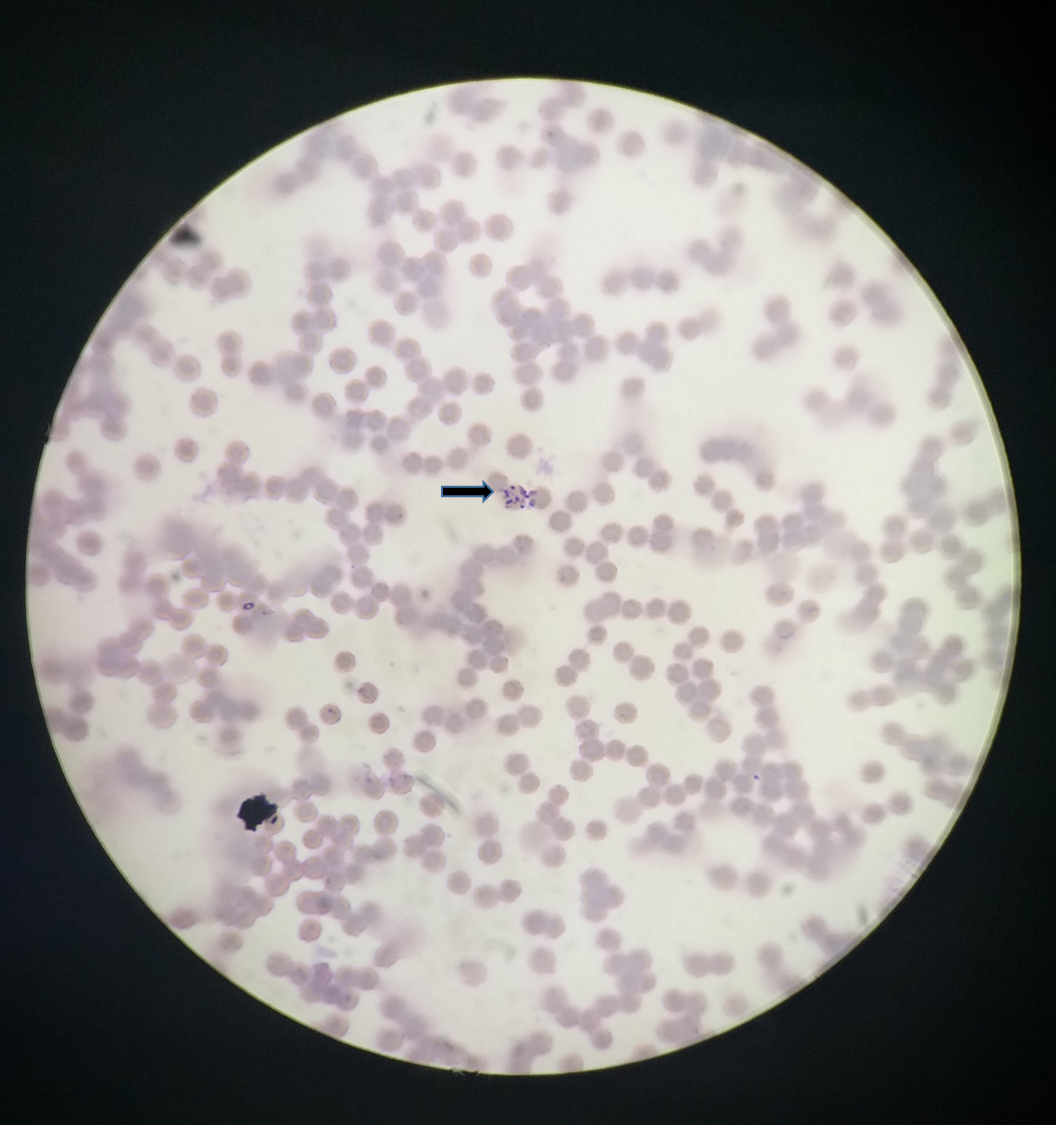


**Supplementary Figure 1.** Monitoring parasitemia in culture of *P. berghei* via thin blood smears staining with Giemsa stain


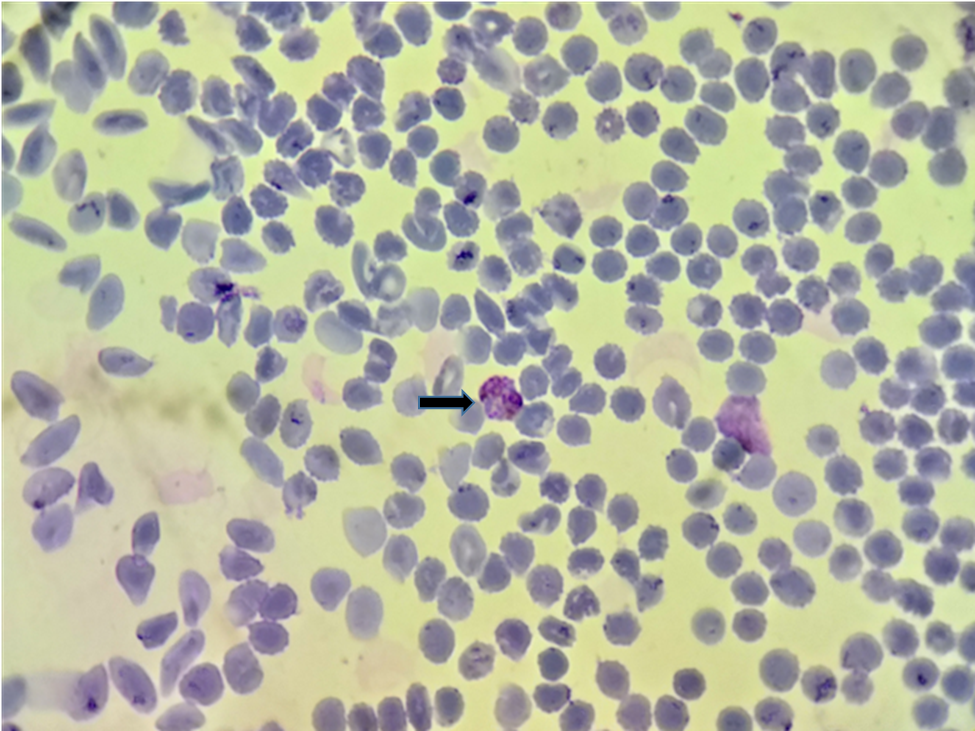


**Supplementary Figure 2.** Monitoring parasitemia in culture of *P. berghei* via thin blood smears staining with Giemsa stain
